# Concurrent congenital diaphragmatic hernia and Hirschsprung’s disease: a diagnostic dilemma with therapeutic implications—a case report and literature review

**DOI:** 10.1093/jscr/rjaf713

**Published:** 2025-09-05

**Authors:** Yasmine Houas, Yasmine Karoui, Hela Oueslati, Nada Sghairoun, Riadh Jouini

**Affiliations:** Pediatric Surgery Department A, Children Hospital Bechir Hamza, 167, 9th of April Boulevard, Tunis, Tunisia; Tunis El Manar University, 94 Taher Ben Ammar Avenue, Romana 1068, Tunisia; Pediatric Surgery Department A, Children Hospital Bechir Hamza, 167, 9th of April Boulevard, Tunis, Tunisia; Tunis El Manar University, 94 Taher Ben Ammar Avenue, Romana 1068, Tunisia; Pediatric Surgery Department A, Children Hospital Bechir Hamza, 167, 9th of April Boulevard, Tunis, Tunisia; Tunis El Manar University, 94 Taher Ben Ammar Avenue, Romana 1068, Tunisia; Pediatric Surgery Department A, Children Hospital Bechir Hamza, 167, 9th of April Boulevard, Tunis, Tunisia; Tunis El Manar University, 94 Taher Ben Ammar Avenue, Romana 1068, Tunisia; Pediatric Surgery Department A, Children Hospital Bechir Hamza, 167, 9th of April Boulevard, Tunis, Tunisia; Tunis El Manar University, 94 Taher Ben Ammar Avenue, Romana 1068, Tunisia

**Keywords:** congenital diaphragmatic hernia, Hirschsprung's disease, neonatal surgery, diagnostic delay, case report

## Abstract

We report a case of concurrent congenital diaphragmatic hernia (CDH) and Hirschsprung's disease (HD) in a neonate without syndromic features, representing one of fewer documented cases worldwide. The patient presented with classic CDH symptoms but developed persistent bowel obstruction post-repair, leading to delayed HD diagnosis 4 weeks later. This case highlights the diagnostic challenges in differentiating postoperative ileus from underlying HD in CDH patients.

## Introduction

Congenital diaphragmatic hernia (CDH) is a developmental anomaly characterized by a defect in the diaphragm that allows abdominal viscera to herniate into the thoracic cavity, leading to potentially severe pulmonary hypoplasia and respiratory compromise [1]. Hirschsprung’s disease (HD), on the other hand, is a neurocristopathy resulting from the failure of enteric neural crest cell migration, causing aganglionosis in the distal intestine and functional bowel obstruction [2]. While both conditions are well-described individually, their co-occurrence in the same patient is exceptionally rare, with only a handful of cases reported in the literature. The diagnostic and therapeutic challenges posed by this dual pathology are substantial.

This rarity makes their association difficult to suspect clinically, where CDH typically dominates the clinical presentation with respiratory distress. Herein, we report a case of an association of CDH and HD to highlight the importance of maintaining a high index of suspicion for this rare association, particularly in patients with atypical postoperative courses.

## Case presentation

**Figure 1:**
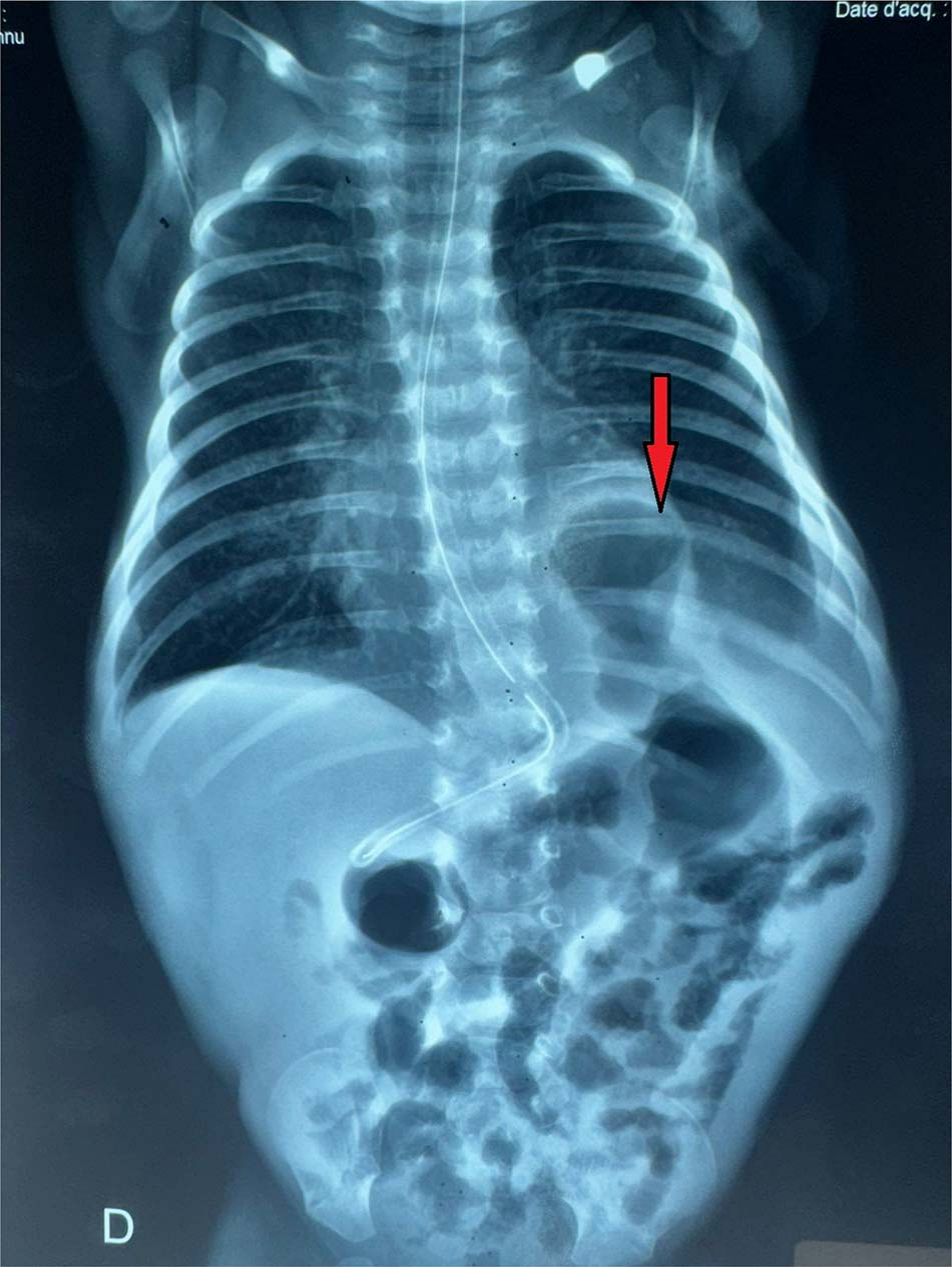
Thoracoabdominal radiograph showing air-filled bowel loops within the thoracic cavity (arrow) with absent visualization of the diaphragmatic contour, consistent with a CDH.

**Figure 2:**
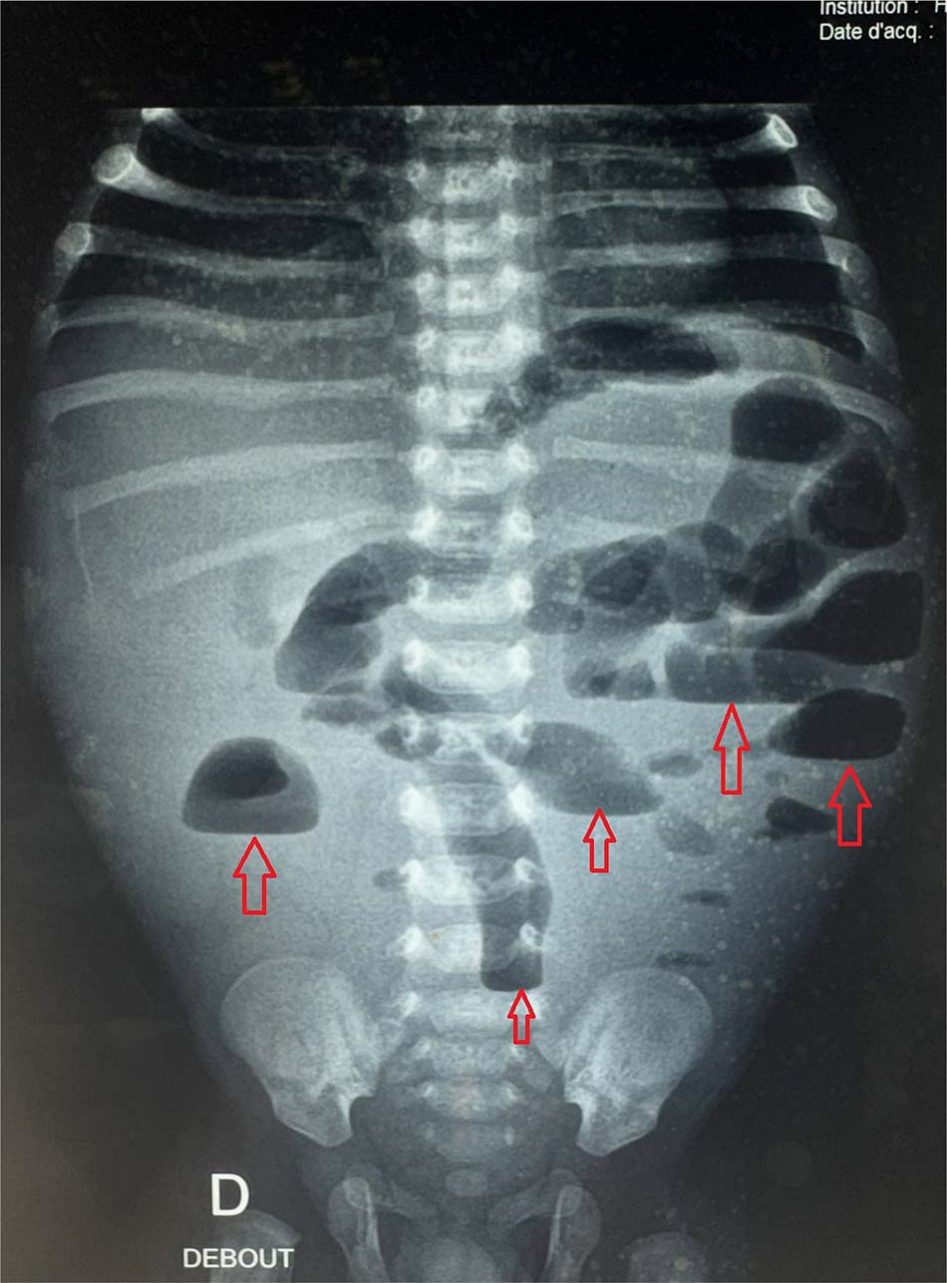
Postoperative abdominal radiograph demonstrating multiple air-fluid levels (arrows) without evidence of significant colonic dilatation.

A male neonate was admitted at 47 h of life with signs of severe respiratory distress requiring immediate intubation. Physical examination revealed a distended abdomen, a prominent thoracic contour, and diminished breath sounds on the left side. A thoraco-abdominal X-ray showed multiple air-filled intestinal loops in the left hemithorax, consistent with a CDH. The patient underwent a primary repair of surgical repair of his diaphragmatic defect (Fig. 1). The initial postoperative course was complicated by multiple episodes of bowel obstruction. Postoperative radiographs demonstrated multiple air-fluid levels without evidence of significant colonic dilatation (Fig. 2). The patient underwent reoperations on postoperative days 21 and 50. Intraoperative findings revealed early adhesions without evidence of other anomalies. Adhesiolysis was performed on both occasions. The patient was discharged on postoperative day 60. At 3 months of age, the child was readmitted with signs of recurrent intestinal obstruction, including bilious vomiting, absence of stool and gas passage, and abdominal distension. The mother reported that spontaneous bowel movements occurred only after the administration of suppositories. Additionally, the child exhibited failure to thrive, with a weight below −2 standard deviations for age. The child had no facial dysmorphism or abnormalities of the limbs or nails.

**Figure 3:**
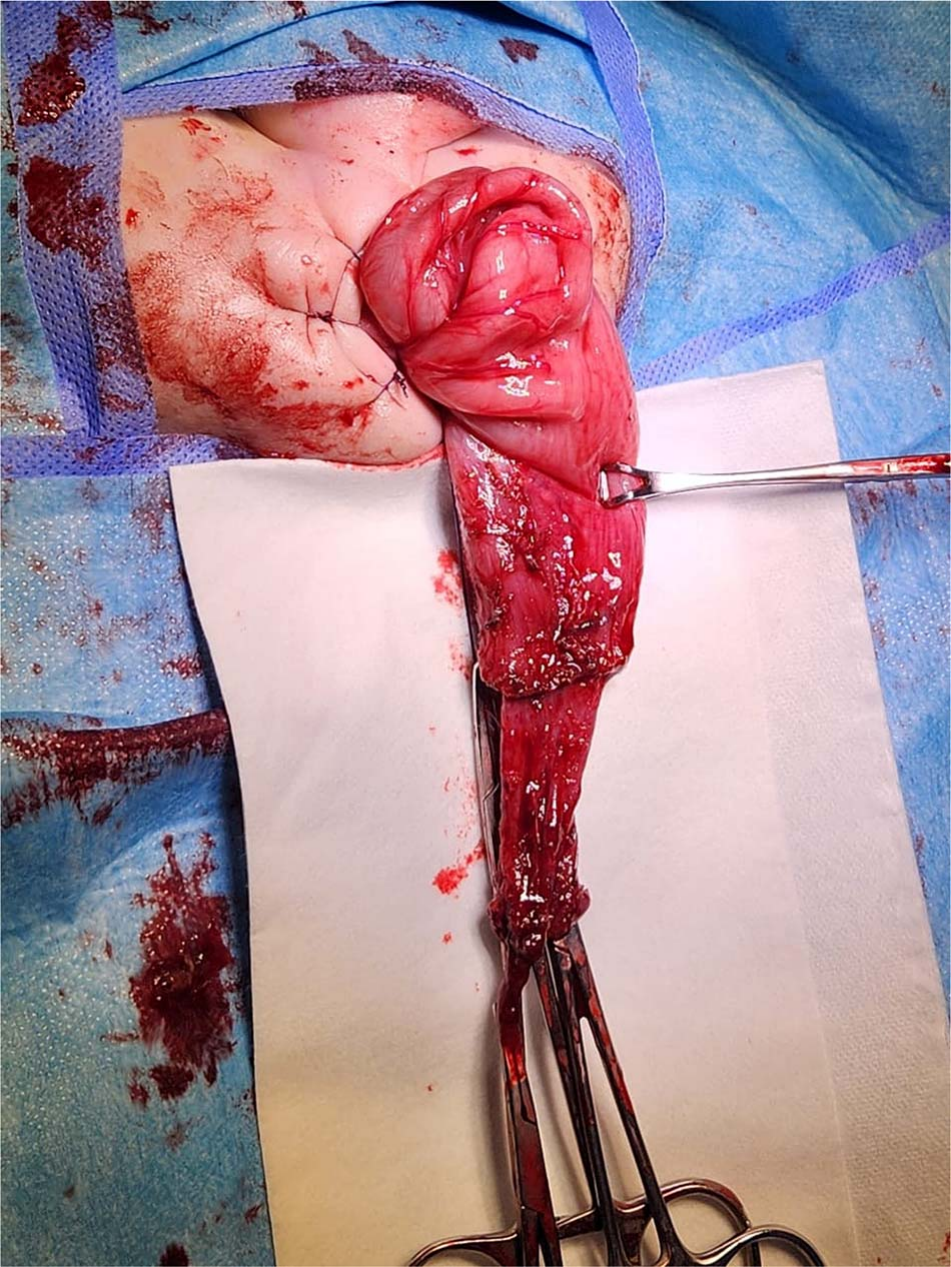
Intraoperative view of a transanal endorectal pull-through (soave procedure) for HD.

Given the clinical presentation and history of recurrent obstruction, HD was suspected. A contrast enema demonstrated a significant caliber discrepancy between a moderately dilated rectum and a markedly dilated sigmoid colon, suggesting a transition zone. A rectal suction biopsy using Noblett forceps confirmed the absence of ganglion cells in the distal bowel. The patient underwent a Soave endoanal pull-through procedure at 4 months of age (Fig. 3). Histopathological analysis of the resected segment confirmed that the pull-through procedure had been performed in a normally ganglionated area. The postoperative recovery was uneventful. The child is currently 3 years old, with normal bowel function and no need for laxatives or enemas. There are no associated congenital anomalies, and the genetic evaluation revealed no abnormalities.

## Discussion

This case report highlights an exceptionally rare association between HD and CDH, with only three confirmed living cases and one postmortem case [3] documented in the medical literature (Table 1).

**Table 1:** Literature review of the living published cases of the association between HD and CDH.

**Authors**	**Age at diagnosis of HD**	**Clinical signs revealing HD**	**Genetic investigation**	**Outcome**
Lenkov *et al*. [4]	12 weeks	Gastric feeds not tolerated	A missense mutation in the FOXP1 gene	Dandy-walker syndrome Feeding via gastrostomy
Nchinda *et al*. [5]	30 days	Increasing bouts of emesis	Heterozygous pathologic variant in the RET^a^ gene	Feeding via gastrostomy
Davis *et al*. [6]	6 days	Postoperative abdominal distension	Fryns syndrome	Mental delay

^a^RET gene: rearranged during transfection.

Notably, all previously reported cases occurred in syndromic patients with identified genetic abnormalities, most frequently Fryns syndrome – a condition characterized by CDH, distinctive facial features, limb anomalies, and multiple organ malformations [7]. Our patient represents the first reported case of this dual pathology occurring in a non-syndromic patient without an identifiable genetic cause, making this presentation particularly unique. The absence of syndromic features in our patient suggests that the co-occurrence of CDH and HD may not always stem from a known genetic syndrome, and raises important questions about potential shared developmental pathways that warrant further investigation.

The clinical presentation in these cases is invariably dominated by the respiratory distress characteristic of CDH, which often leads to delayed diagnosis of the concomitant HD. As demonstrated by our patient's course and supported by the literature review in Table 1, postoperative warning signs such as feeding intolerance, abdominal distension, or failure to thrive should raise strong suspicion for underlying HD. Our patient unfortunately underwent multiple surgical interventions before the correct diagnosis was established, a scenario that could potentially have been avoided with earlier consideration of this rare association.

This diagnostic challenge extends beyond CDH to other congenital digestive anomalies including anorectal malformations [8], intestinal malrotation [9], and intestinal atresias [10]. In all these conditions, any atypical postoperative course featuring persistent obstruction, delayed meconium passage, or recurrent enterocolitis should prompt evaluation for HD. A low threshold for performing rectal biopsies and contrast studies in such cases could lead to earlier diagnosis and improved outcomes.

## Conclusion

This case highlights the crucial need for clinical vigilance regarding the rare but serious association between CDH and HD. Clinicians must maintain a high index of suspicion for concurrent HD when encountering postoperative gastrointestinal complications. The diagnostic challenge is particularly significant given the symptom overlap with normal postoperative recovery and the potential absence of syndromic features in non-genetic cases. Our experience underscores the importance of considering HD in the differential diagnosis of any CDH patient with an atypical recovery course, and suggests that a low threshold for rectal biopsy may be warranted in these complex cases.
